# Revisiting the Karyotype Evolution of Neotropical Boid Snakes: A Puzzle Mediated by Chromosomal Fissions

**DOI:** 10.3390/cells9102268

**Published:** 2020-10-10

**Authors:** Patrik F. Viana, Tariq Ezaz, Marcelo de Bello Cioffi, Thomas Liehr, Ahmed Al-Rikabi, Rodrigo Tavares-Pinheiro, Luiz Antônio Carlos Bertollo, Eliana Feldberg

**Affiliations:** 1 Laboratory of Animal Genetics, Instituto Nacional de Pesquisas da Amazônia, Coordenação de Biodiversidade, Av. André Araújo 2936, Petrópolis, Manaus 69067-375, AM, Brazil; Patrik.biologia@gmail.com (P.F.V.); feldberg@inpa.gov.br (E.F.); 2 Institute for Applied Ecology, Faculty of Science and Technology, University of Canberra, Canberra 12 2616, ACT, Australia; Tariq.Ezaz@canberra.edu.au; 3 Departamento de Genética e Evolução, Universidade Federal de São Carlos, São Carlos 13565-090, SP, Brazil; mbcioffi@ufscar.br (M.d.B.C.); bertollo@ufscar.br (L.A.C.B.); 4 Institute of Human Genetics, University Hospital Jena, Am Klinikum 1, 07747 Jena, Germany; ahmedgenetic@hotmail.com; 5 Departamento de Ciências Biológicas e da Saúde, Laboratório de Herpetologia, Universidade Federal do Amapá, Macapá 68903-419, AP, Brazil; rodrigotmcp@gmail.com

**Keywords:** chromosomal painting, evolution, neotropical region, Serpentes, Booidea

## Abstract

The Boidae family is an ancient group of snakes widely distributed across the Neotropical region, where several biogeographic events contributed towards shaping their evolution and diversification. Most species of this family have a diploid number composed of 2n = 36; however, among Booidea families, the Boidae stands out by presenting the greatest chromosomal diversity, with 2n ranging between 36 and 44 chromosomes and an undifferentiated XY sex chromosome system. Here, we applied a comparative chromosome analysis using cross-species chromosome paintings in five species representing four Boidae genera, to decipher the evolutionary dynamics of some chromosomes in these Neotropical snakes. Our study included all diploid numbers (2n = 36, 40, and 44) known for this family and our comparative chromosomal mappings point to a strong evolutionary relationship among the genera *Boa*, *Corallus*, *Eunectes*, and *Epicrates*. The results also allowed us to propose the cytogenomic diversification that had occurred in this family: a process mediated by centric fissions, including fission events of the putative and undifferentiated XY sex chromosome system in the 2n = 44 karyotype, which is critical in solving the puzzle of the karyotype evolution of boid snakes.

## 1. Introduction

**Figure 1:**
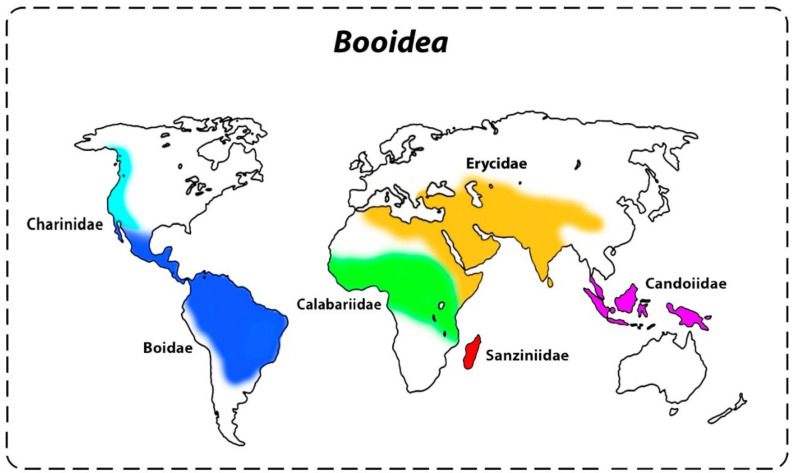
Estimated worldwide distribution pattern of Booidea species according to Reynolds and Henderson [14] and Uetz and Hosek [4].

The modern boas (Booidea) and pythons (Pythonoidea) are an ancient group of snakes, often referred to as primitive snakes (Henophidia). Currently, comprising more than 220 valid species, the former diversified lineages of snakes, jointly with Scolecophidia and Caenophidia, represent the three main groups that make up the Serpentes suborder [1,2,3,4]. The Booidea superfamily is a monophyletic group [5,6], including six families, Calabariidae, Candoiidae, Sanziniidae, Charinidae, Erycidae, and Boidae, with over 66 species that are distinct both morphologically and biogeographically [5,7,8]. Calabariidae is restricted to West Africa, whereas Candoiidae is restricted to the Australasian countries, Sanziniidae to Madagascar, Charinidae to Central and North Americas (excepting for a single Colombian species), Erycidae to Europe, Middle East, North Africa, and Central and Southern Asia, and Boidae to the Neotropical region (Figure 1) [4,5,6,9]. This worldwide distribution is the result of numerous biogeographic events associated with the Gondwana fragmentation [10,11,12,13,14].

The Neotropical Boidae family stands out as an intriguing group due to its stunning and complex evolutionary and biogeographic history dating from the end of the Cretaceous period [12,13,15,16]. Boids are viviparous species inhabiting a wide range of environments and microhabitats, including different forest landscapes that reflect their varied behaviors, such as generalist (*Boa* and *Epicrates*), aquatic (*Eunectes*), and arboreal (*Corallus* and *Chilabothrus*) [9,17,18]. Part of the diversification of these snakes was also mediated by vicariant events (e.g., marine incursions, uplift of the Andes, and geology of the Isthmus of Panama) [6,16,19,20,21]. Boidae is also the most speciose group inside the Booidea superfamily, comprising more than 30 species allocated in five genera, *Boa*, *Corallus*, *Eunectes*, *Epicrates*, and *Chilabothrus* [4,5,6], representing the Neotropical radiation from this superfamily [5,9,15]. These snakes differ in biogeographic, morphological, molecular, and even chromosomal characteristics [5,6,22,23,24].

Most of the Serpentes lineages have 2n = 36 chromosomes (16 macro + 20 microchromosomes), which is considered a plesiomorphic feature for the suborder [25,26,27,28]. However, Boidae differs from other families by presenting the greatest chromosomal diversity among the Booidea families, with 2n ranging from 36 to 44 chromosomes, including an undifferentiated XY sex chromosome system [24,25,29]. Robertsonian and non-Robertsonian rearrangements, including fission events [26], have often been proposed to explain this karyotype diversity. Furthermore, the occurrence of interstitial telomeric sequences (ITSs) in most of the analyzed species strongly suggests that multiple chromosomal rearrangements contributed to such diversity [24,30].

Almost six decades ago, the first steps towards understanding the cytotaxonomy of the Neotropical snakes were taken by Professor Willy Beçak, a Brazilian pioneer researcher trying to unravel their chromosome evolution. Beçak discovered a remarkable karyotype diversity for Neotropical Boidae snakes, finding 2n = 36 chromosomes for three genera (*Boa*, *Eunectes*, and *Epicrates*) and 2n = 44 chromosomes in the *Corallus caninus* species, whose karyotype was composed only of subtelo/acrocentric like chromosomes. However, the lack of refined taxonomic/molecular tools at the time led Beçak to conclude that 2n = 44 could represent the plesiomorphic condition for the Boidae family and that 2n = 36 would be a derived condition originating from chromosomal fusions.

Years later, Gorman and Gress [26] added another key piece to this puzzle, by identifying the 2n = 40 for the species *Corallus grenadensis* from the West Indies, an intermediate 2n between the 36 as found in *Boa*, *Eunectes*, *Epicrates*, and *Chilabothrus* and 44, present in *Corallus caninus*. This intermediate 2n configuration led Gorman and Gress [26] to suggest that the evolution of these snakes had been driven by chromosomal fissions instead of fusions, as previously proposed by Beçak [25], especially because the genus *Chilabothrus* (with 2n = 36 and nominally considered *Epicrates* at the time) was suggested as an older lineage than *Corallus*. However, the interpretation that fission events mediated the karyotype evolution of boid snakes was due to a sum of coincidences, since *Chilabothrus* species, although possessing 2n = 36, are not the older lineages in the Boidae family [5,6], but instead *Boa*, which also has 2n = 36 chromosomes.

Given both scenarios of karyotype evolution proposed for neotropical boids, we carried out a cross-species chromosomal investigation performing chromosomal paintings in karyotypes of different Boidae species to decipher events of chromosome rearrangements associated with the evolutionary processes and diversification of this group. Cross-species chromosomal painting is a valuable comparative genomic tool in accessing and deciphering the dynamics of karyotype and genome evolution [31,32,33]. In this way, we used representative species of the genera *Boa*, *Corallus*, *Eunectes*, and *Epicrates*, covering all diploid numbers (2n = 36, 40 and 44) known for the family. Analysis of our data enabled us to estimate the ancestral diploid number for the family, allowing us to shed light on the puzzle of the evolution of Boidae karyotypes.

## 2. Material and Methods

### 2.1. Sampling and Mitotic Chromosome Preparation

Adult snakes were collected from natural populations across the Amazon region under permission granted by Instituto Chico Mendes de Conservação da Biodiversidade (ICMBio), number: 45275. Chromosomes of *Boa constrictor* (4 ♂ and 4 ♀; 2n = 36) (Amazonian red-tailed boa), *Corallus hortulana* (2 ♂ and 2 ♀; 2n = 40) (Amazon tree boa), *Corallus caninus* (2 ♂ and 2 ♀; 2n = 44) (Negro basin emerald tree boa), *Eunectes deschauenseei* (2 ♂ and 3 ♀; 2n = 36) (Dark-spotted anaconda), and *Epicrates maurus* (3 ♂ and 2 ♀; 2n = 36) (North rainbow boa) were obtained from small blood samples cultured for 4 days at 29 °C, following the protocol detailed by Viana et al. [34]. gDNA from each individual was extracted from blood using the Wizard Genomic Purification Kit (Promega), according to the manufacturer’s recommendations. It is highlighted that no animals were euthanized in this study. All procedures and experimental protocols in this study were approved, performed in accordance with all relevant guidelines and regulations, and fulfill the rules of the Ethics Committee of the National Institute of Amazonian Research (permission number: 018/2017).

### 2.2. Microdissection and Chromosome Painting Probe Preparation

Fifteen copies of the first four chromosome pairs (1m, 2sm, 3m, and 4m) of *Boa constrictor* were isolated via microdissection and amplified following Yang et al. [35]. The whole chromosome-derived probes (designated as Bc1, Bc2, Bc3, and Bc4) were labeled with Spectrum Orange-dUTP or Spectrum Green-dUTP (Vysis, Downers Grove, IL, USA) through 30 cycles of secondary DOP PCR, using 1 μL of the primary amplification product as a template DNA, resulting in a final volume of 20 μL [35]. The final probe cocktail for each slide was composed of 500 ng of the probes and 60 µg of Cot-1 DNA isolated from the *Boa constrictor* total genomic DNA (for details, see Zwick et al. [36]), to outcompete the hybridization of highly-repeated DNA sequences. However, experiments conducted without Cot-1 DNA as a competitor produced the same results. Hybridization procedure followed our previous studies [37,38,39,40] and was performed for 1 day (24 h) in *Boa constrictor* and 3 days (72 h) for all the other species, at 37 °C in a dark and moist chamber. After washing procedures, the chromosomes were counterstained with DAPI (1.2 µg/mL) and mounted in antifade solution (Vector).

### 2.3. Microscopy and Image Analyses

At least 10 metaphase spreads per individual, for each probe, were analyzed to confirm the karyotype structure and FISH results. Images were captured using an Olympus BX51 microscope (Olympus Corporation, Ishikawa, Japan). Chromosomes were classified as metacentric (m), subtelocentric (st), or acrocentric (a), according to their arm ratios [41].

## 3. Results

**Figure 2:**
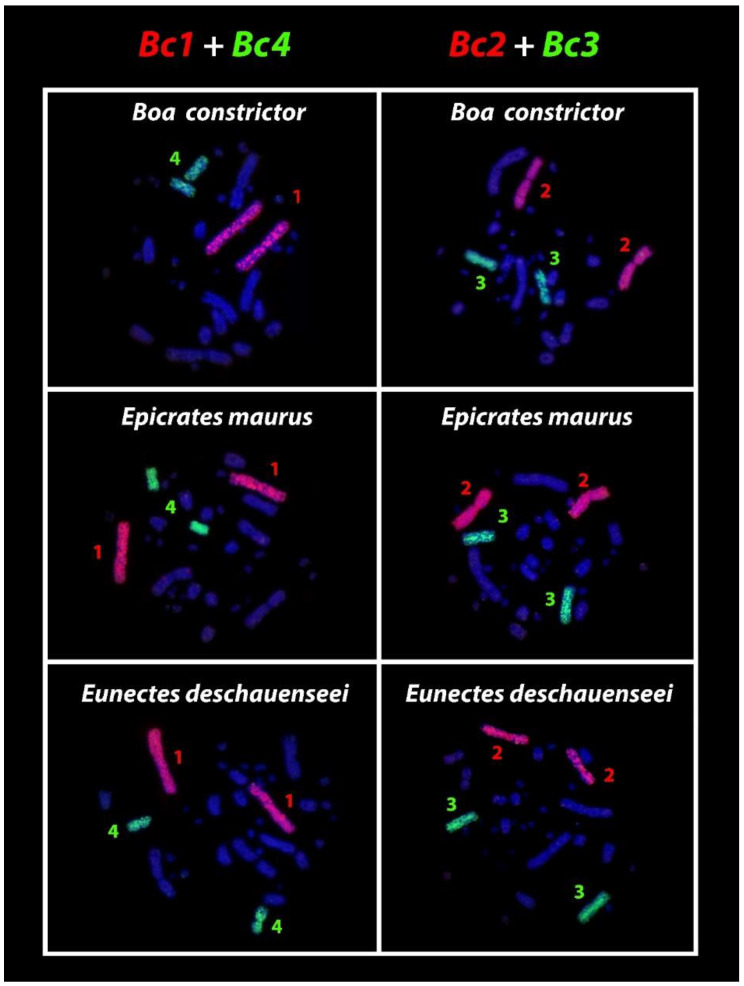
Whole chromosome painting using the Bc1, Bc2, Bc3, and Bc4 *Boa constrictor* derived probes in chromosomal backgrounds of *Boa constrictor* (2n = 36), *Epicrates maurus* (2n = 36), and *Eunectes deschauenseei* (2n = 36). Note the full homologies of the four chromosome pairs in all of these species. Because the hybridization patterns for males and females were exactly the same, we selected representative metaphase to illustrate the results.

The Amazonian red-tailed boa (*Boa constrictor*), the dark-spotted anaconda (*Eunectes deschauenseei*) and the North rainbow boa (*Epicrates maurus*) have 2n = 36 chromosomes (16 macro + 20 microchromosomes), similar to other karyotyped *Boa*, *Eunectes*, and *Epicrates* species [24]. These three species also share full chromosomal homologies with the Bc1, Bc2, Bc3, and Bc4 probes (Figure 2), evidencing synteny among their karyotypes.

**Figure 3:**
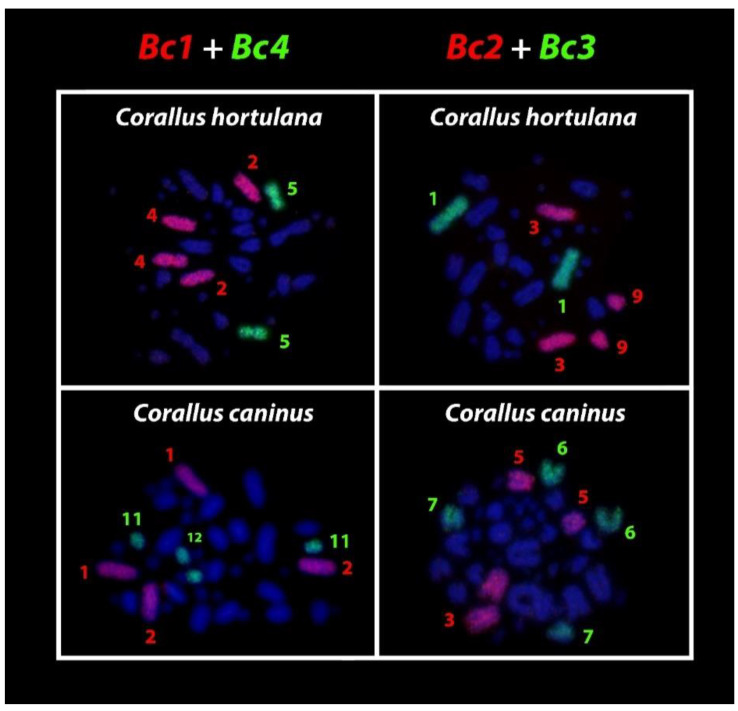
Chromosomal painting using the Bc1, Bc2, Bc3, and Bc4 *Boa constrictor* (2n = 36) derived probes in the chromosomal backgrounds of *Corallus hortulana* (2n = 40) and *Corallus caninus* (2n = 44). Note that despite their divergent diploid numbers, both species still share chromosome homologies with the oldest genus (*Boa*) of the Boidae family. Likewise, because the hybridization patterns for males and females were the same, we also selected representative metaphase to illustrate the results.

*Corallus hortulana* from Central Amazonia (Amazon tree boa) has 2n = 40 chromosomes (4m + 16st/a + 20 microchromosomes), corroborating previously published data [24]. This species differs from the other Boidae by having a smaller number of metacentric chromosomes and a greater number of subtelo/acrocentric ones [24]. Bc1 corresponds to chromosome pairs 2 and 4 (st/a) and Bc2 to pairs 3 and 9 (st/a), while Bc3 and Bc4 are found intact as metacentric chromosome pairs 1 and 5, respectively (Figure 3).

*Corallus caninus* has 2n = 44 chromosomes, 24 macrochromosomes predominantly subtelo/acrocentrics, and 20 microchromosomes, similar to the pattern described by Beçak [42]. Similar to the Amazon tree boa, *C. caninus* also shares chromosomal homologies with Bc1, Bc2, Bc3, and Bc4 *Boa constrictor* pairs. Bc1 corresponds to the chromosome pairs 1 and 2, Bc2 to pairs 3 and 5, Bc3 to pairs 6 and 7, and Bc4 to pairs 11 and 12 (Figure 3). We highlight that there were no differences among individuals or between males and females in the patterns observed within all species.

## 4. Discussion

During the past few decades, several studies have attempted to unravel the evolutionary patterns of diversification and phylogenetic relationships of the Neotropical boid lineages [6,12,16], despite an evident cryptic genetic and morphological diversity present in this ancient snake group [22,23,43,44]. Today, these animals possess relatively well-resolved phylogenetic relationships, recovering *Boa constrictor* (i.e., the oldest lineage of the Boidae family) as the sister group of the clade composed of *Corallus*, *Eunectes*, *Epicrates*, and *Chilabothrus* genera [5,6,12,16,45].

**Figure 4:**
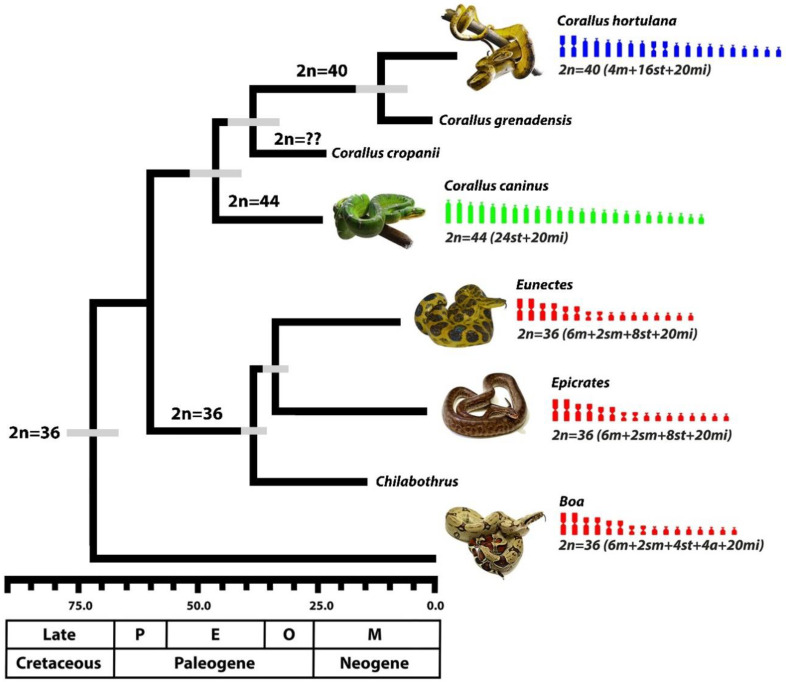
Chronogram of Neotropical boid snakes, adapted from Colston et al. [16]. Only macrochromosomes are illustrated, since all species retain 20 microchromosomes. Highlighted in blue, the 2n = 40; green, 2n = 44; and in red, the 2n = 36. P = Paleocene; E = Eocene; O = Oligocene; M = Miocene. We highlight that the tree proposed by Colston et al. [16] displays the phylogenetic relationships between species and does not represent the current age of the chromosomal rearrangement’s events.

Currently, three diploid numbers are known for Boid snakes, 36, 40, and 44, and our comparative chromosomal mappings point to a strong evolutionary relationship among the genera *Boa*, *Corallus*, *Eunectes,* and *Epicrates*. The chromosome pairs 1, 2, 3, and 4 of *Boa constrictor* (2n = 36) are the same as observed in the *Eunectes* and *Epicrates* genera (2n = 36) (Figure 2). This suggests that this karyotype configuration (2n = 36) was already present in the most recent common ancestor that gave rise to the genus *Boa* at the end of the Cretaceous ~75 mya and retained in *Eunectes*, *Epicrates*, and *Chilabothrus* (Figure 4).

**Figure 5:**
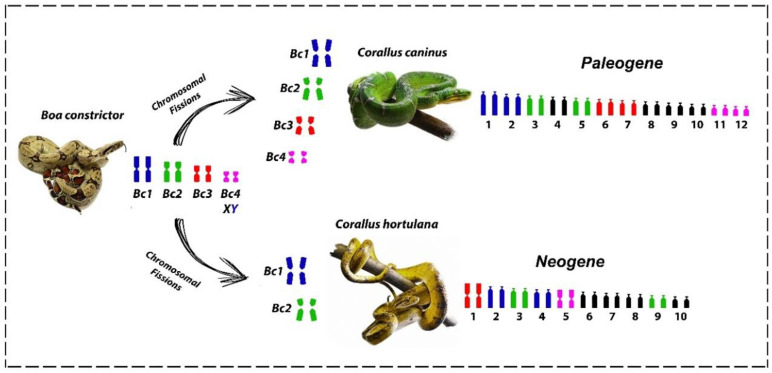
Schematic representation of the macrochromosome diversification in *Corallus* species mediated by chromosomal fissions. The first four chromosome pairs of *Boa* constrictor correspond to 8 pairs in *Corallus caninus* and 6 pairs in *Corallus hortulana* (highlighted by the colors).

However, these chromosome pairs underwent centric fissions and can also be found in independent diversification events spanning ~60 mya (Paleogene/Neogene), which gave rise to the modern tree boa species of the genus *Corallus* in the Neotropical region (Figure 4). The sum of the available data indicates that the karyotype diversification of the *Corallus* genus occurred through chromosomal fissions in the first four pairs of the common ancestral complement for the Boidae family (pairs 1m, 2sm, 3m, 4m), as found in *Boa constrictor* (Figure 5). However, additional fission/fusion events that may have taken place (e.g., involving the acrocentrics or even microchromosomes) cannot be ruled out.

**Figure 6:**
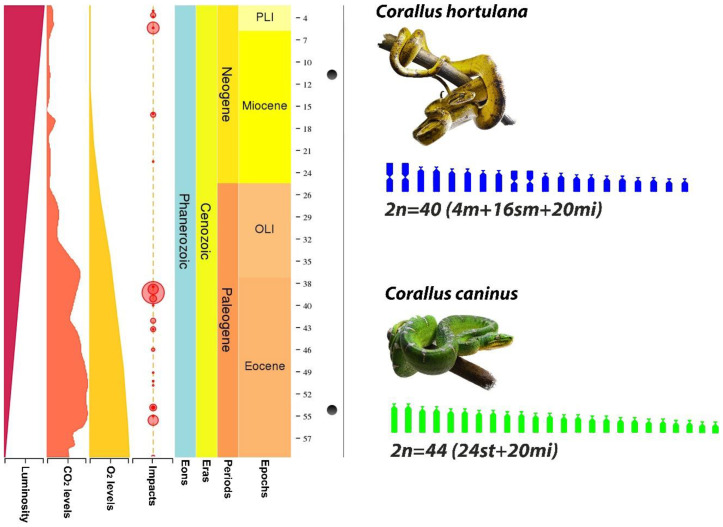
Estimated time of partial divergence between 2n = 40 (*Corallus hortulana*—Neogene) and 2n = 44 (*Corallus caninus*—Paleogene), with 45 million years of split and independent evolution on average. Graphics indicate the impacts, O_2_ levels, CO_2_ levels, and luminosity conditions on Earth in each geological scale, along with the species diversification in the Neotropical region. The estimated pairwise was retrieved from the TimeTree online database (http://www.timetree.org/).

The karyotype diversification in *Corallus* species, interestingly, did not follow the usual evolutionary trend, from 2n = 36 to 2n = 40 to 2n = 44, as suggested by Gorman and Gress [26]. Indeed, *Corallus* genus (2n = 40 and 44) and its sister group, the clade *Eunectes*, *Epicrates,* and *Chilabothrus* (all species with 2n = 36), shared the most recent common ancestor dating back to the early Paleogene (Figure 4). The *Corallus* lineages exhibit two karyotype configurations, the 2n = 40 and 2n = 44 chromosomes that arose in the middle of the Paleogene/Eocene from centric fissions in all meta and submetacentric chromosomes (pairs 1m, 2sm, 3m, and 4m), which are present in their most recent common ancestor (Figure 4 and Figure 5). *Corallus* experienced different evolutionary pressures along with their diversification in the Neotropical region (Figure 6), following different evolutionary trajectories, especially if we take into account that the 2n = 44 composed only of subtelo/acrocentric chromosomes is exclusively found in *Corallus caninus*. However, the 2n = 40 is retained during the diversification events of the other Neotropical tree boa species, such as the split of the clade composed of *Corallus hortulana* and *Corallus grenadensis*, which dates back to recent ~10 mya in the Neogene (Figure 4). Meanwhile, the ancestral state 2n = 36 is retained along the diversification and split among *Eunectes*, *Epicrates*, and *Chilabothrus* genera in the Paleogene/Neogene (Figure 4).

Interestingly, the undifferentiated putative XY sex chromosomes of *Boa constrictor* [29,46] remained conserved in species with 2n = 36 (putative pair 4 in *Eunectes* and *Epicrates*) and with 2n = 40 (pair 5 in *Corallus hortulana*). On the other hand, in *Corallus caninus* (2n = 44), the putative XY chromosomes of *Boa constrictor* underwent fission events, giving rise to the smallest acrocentric chromosomes of the complement, pairs 11 and 12 (Figure 3 and Figure 5). This chromosomal homology of the putative XY (4th pair), across Boidae lineages, suggests that other genera of the family may also share an undifferentiated XY system of sex chromosomes, even though *Corallus caninus* presents the putative *Boa* XY as four small acrocentric chromosomes (pairs 11 and 12) (Figure 5). Furthermore, consecutive parthenogenetic births in *Eunectes*, *Epicrates* and *Chilabothrus*, producing exclusively females, also support an XY system shared among Boidae species [29,47,48,49]. In *Corallus caninus*, the putative *Boa* XY is homologous to pairs 11 and 12 due to a fission event, however, it is uncertain whether a functional XY sex chromosome system exists in this lineage or if a multiple XY system evolved through this fission event. Comparative mappings from the putative *Boa* XY derived probes in sister lineages, such as the well-differentiated ZW (also the 4th pair) present in *Acrantophis* [50], will certainly highlight the homologies of sequences as well as turnovers between the sex chromosome systems in these ancient groups of snakes.

In conclusion, our study shed light on the puzzle of the karyotype evolution of Neotropical boids. We estimated the likely ancestral karyotype status for the Boidae family and detailed the cytogenomic diversification that had occurred in this family. Particularly, our study highlighted that processes mediated by fissions generated the current karyotype configurations of this Neotropical clade of primitive snakes. This study is part of a series of further cytogenetic and genomic studies focusing on Neotropical reptiles and their hidden evolutionary diversity.
